# Validity and utility of the Japanese version of the brief unhelpful thoughts and beliefs about stuttering scale: UTBAS-6-J

**DOI:** 10.3389/fpsyg.2024.1382673

**Published:** 2024-06-11

**Authors:** Shuta Tomisato, Yasuto Yada, Koichiro Wasano, Takeyuki Kono, Hiroyuki Ozawa

**Affiliations:** ^1^Department of Otorhinolaryngology, Head and Neck Surgery, Keio University School of Medicine, Tokyo, Japan; ^2^Department of Otolaryngology, Nippon Koukan Hospital, Kawasaki, Kanagawa, Japan; ^3^Department of Language Sciences, Tokyo Metropolitan University, Tokyo, Japan; ^4^Department of Otolaryngology, Head and Neck Surgery, Tokai University School of Medicine, Isehara, Kanagawa, Japan

**Keywords:** stuttering, questionnaire, social anxiety disorder, UTBAS-6, Japanese speaker

## Abstract

Do adults who stutter have abnormally high social anxiety? Is it related to maladaptive cognition? As these are persistent, unresolved questions in stuttering research, it behooves clinicians to at least assess and attempt to identify social anxiety in patients who stutter and its basis before decisions are made about stuttering treatment. The Unhelpful Thoughts and Beliefs About Stuttering (UTBAS) scale is a self-administered questionnaire that measures the degree of non-adaptive cognition in people who stutter (PWS) due to social anxiety. The 66-item UTBAS is time-consuming to complete, prompting the development of a shorter 6-item version, the UTBAS-6, which is in English. Here, we aimed to assess some psychometric properties of the Japanese version of the UTBAS-6, the UTBAS-6-J, which has not been done to date. In 56 adult patients (mean 32.6 ± 11.1 years) who stutter, we quantified the reliability, the internal consistency, and the concurrent validity of the UTBAS-6-J. Along with the UTBAS-6-J, patients also were administered the Overall Assessment of the Speaker’s Experience of Stuttering – Japanese version (OASES-A-J), the Modified Erickson Communication Attitude Scale – Japanese version (S-24-J), and the Liebowitz Social Anxiety Scale – Japanese version (LSAS-J). Cronbach’s alpha for UTBAS-6-J total scores was 0.974, indicating excellent internal consistency. UTBAS-6-J scores were significantly correlated with scores on the OASES-A-J, the S-24-J, and the LSAS-J (all *p* < 0.005). Concurrent validity of the UTBAS-6-J with these three questionnaires was confirmed. The UTBAS-6-J has good internal consistency and concurrent validity, which will aid clinical decision-making about stuttering treatments.

## 1 Introduction

Stuttering is a disorder of speech fluency that can severely interfere with effective communication. Accumulating evidence points to stuttering as being strongly associated with social anxiety (Stein et al., 1996; Schneier et al., 1997; Mulcahy et al., 2008; Blumgart et al., 2010; Craig and Tran, 2014; Iverach et al., 2016b). People who stutter (PWS) are six to seven times more likely than control subjects to meet diagnostic criteria for social anxiety disorder (Iverach et al., 2009), and recent estimates suggest that 40% of PWS meet the diagnostic criteria for social anxiety disorder (Blumgart et al., 2010). For Japanese speakers, social anxiety and stuttering are also related. For example, Japanese adults who stutter tend to experience higher levels of social anxiety than those who do not stutter, according to the results of the screening tests for social anxiety disorder (Chu et al., 2020; Tomisato et al., 2022). Although several studies have shown that the degree of social anxiety does not correlate with the severity of objective stuttering symptoms (e.g., Iverach et al., 2018; Tomisato et al., 2022), it is imperative in clinical practice to recognize that stuttering is not always a stand-alone disorder. Rather, physicians need to consider the relationship between stuttering and social anxiety, and avoid focusing solely on the superficial symptoms of stuttering, especially when considering treatment options.

People who suffer from social anxiety disorder frequently have non-adaptive thoughts, which play a persistent role in the maintenance of social anxiety disorder (Clark and Wells, 1995; Foa et al., 1996; Kocovski et al., 2011). Thus, when providing treatments like cognitive behavioral therapy (CBT), therapists must be aware of and acknowledge possible non-adaptive thoughts of clients with social anxiety disorder. Non-adaptive thoughts also contribute to the maintenance of social anxiety disorder in stuttering (Iverach et al., 2017). Thus, it is critical for therapists not to dismiss their clients’ non-adaptive thoughts as being unrelated but instead to try to understand them and their possible contribution to stuttering.

The Unhelpful Thoughts and Beliefs About Stuttering (UTBAS) scale was developed as a self-administered instrument to measure non-adaptive thoughts related to stuttering and social anxiety in adults seeking treatment in Australia (St Clare et al., 2009). UTBAS items were developed using a comprehensive file audit of adults who sought treatment for stuttering over a 10-year period (St Clare et al., 2009). The original 66-item UTBAS measures only how frequently PWS think about these non-adaptive thoughts about stuttering. Both the clinical utility and the validity of the UTBAS were confirmed after adding “how much you believe” and “how anxious you are,” in addition to the negative thoughts (Iverach et al., 2011). For each of the 66 items of the UTBAS, PWS are asked to rate on a 5-point scale (e.g., 1 = never or not at all; 5 = always or totally) to what extent they experience the following: (I) how frequently they have negative thoughts; (II) how much they believe these thoughts; and (III) how anxious these thoughts make them feel. Next, for each of the 66 items, the scores for each of these three areas are summed to arrive at total subscores for I, II, and III. Finally, these subscores are summed to obtain the total UTBAS score.

The UTBAS has been translated into Japanese (UTBAS-J; Chu et al., 2017) using the standard forward-backward translation process (Brislin, 1970; Ann, 2016), and evaluated for its psychometric properties (Chu et al., 2017). The UTBAS-J has good test–retest reliability and high internal consistency (Chu et al., 2017), making it a promising assessment tool for PWS in clinical and research settings.

However, the UTBAS requires three responses for each of its 66 items of anxiety being assessed (i.e., 198 responses in total) (Iverach et al., 2011). A more compact version of the UTBAS would be helpful for screening purposes because the full version is too laborious and cumbersome to administer and use in clinical settings. Therefore, a brief 6-item version (UTBAS-6) was developed (Iverach et al., 2016a). The goal of this brief version was to save time, while maintaining the reliability and validity of the original UTBAS. Like the UTBAS, the UTBAS-6 contains three inquiries for each of its items: “how frequently I have these thoughts,” “how much I believe these thoughts,” and “how anxious these thoughts make me feel” (Iverach et al., 2016a).

As with the original UTBAS, the UTBAS-J requires subjects to make three responses for its 66 items (i.e., 198 responses in total) and is thus time-consuming and burdensome to use in clinical settings; the UTBAS-J takes 40–60 min to complete (Chu et al., 2017). Thus, we sought to produce a brief Japanese-language version of the UTBAS-6, the UTBAS-6-J, with the goal of using it as a screening tool that can be quickly completed, scored, and evaluated during a doctor’s office visit. Ideally, the UTBAS-6-J would be comparable to the English UTBAS-6 (except in Japanese) and would contain the same items as the UTBAS-6. One goal of the present study was to assess the reliability and validity of the UTBAS-6-J, which we expected would be equally reliable and valid as the UTBAS-J and UTBAS-6.

Because stuttering is complex and can present with other problems, such as social anxiety, stuttering needs to be evaluated from various perspectives. Since evaluation tools for assessing stuttering in the Japanese language are largely lacking (Chu et al., 2014), establishing a new Japanese-version evaluation tool specifically for use in clinical practice is a significant milestone. In addition, if the results from using the new tool were to conflict with those of existing assessment tools, clinicians may have difficulty comparing and interpreting the results. Therefore, another objective of this study was to examine the concurrent validity of the UTBAS-6-J in light of other relevant assessment tools. Thus, we administered the UTBAS-6-J alongside the following three questionnaires, which deal with different aspects of stuttering behavior: the Modified Erickson Communication Attitude Scale – Japanese version (S-24-J), a questionnaire that measures communication attitudes and that is widely used in clinical stuttering practice in Japan (Sasanuma and Ito, 1998; Iimura and Ishida, 2022); the Liebowitz Social Anxiety Scale – Japanese version (LSAS-J), a screening test for social anxiety disorder (Asakura et al., 2002); and the Overall Assessment of the Speaker’s Experience of Stuttering – Japanese version (OASES-A-J), a comprehensive assessment tool designed to measure the impact of stuttering (Sakai et al., 2017). This parallel administration of multiple, validated instruments would aid our analysis of concurrent validity.

It is anticipated that scores on the UTBAS-6-J will demonstrate a positive correlation with the aforementioned questionnaires. The rationale for this is as follows. As previously stated, numerous studies have indicated that stuttering is associated with social anxiety (Stein et al., 1996; Schneier et al., 1997; Mulcahy et al., 2008; Blumgart et al., 2010; Craig and Tran, 2014; Iverach et al., 2016b), suggesting the presence of non-adaptive cognitions in the background (Iverach et al., 2017). In addition, communication attitudes have been shown to be associated with social anxiety in PWS (Tomisato et al., 2022). Given the association between social anxiety and non-adaptive cognitions, it is expected that the degree of non-adaptive cognitions will correlate with reluctance to communicate. Indeed, the full version of the UTBAS-J has shown a correlation with the S-24 (Chu et al., 2017). It is therefore anticipated that the “reluctance to communicate attitude (S-24-J),” “social anxiety (LSAS-J),” and “adverse impact of stuttering (OASES-A-J)” will correlate with the “non-adaptive perception of stuttering,” as measured by the UTBAS-6-J.

## 2 Materials and methods

### 2.1 Participants

Participants were 56 PWS who presented to the Nippon Koukan Hospital between January 1, 2018, and May 31, 2019. Of the 56, 46 were males, 10 were females; mean age ± SD was 32.6 ± 11.1 years. Their primary complaint was stuttering. Thus, participants self-selected for this study. All participants were diagnosed with stuttering according to DSM-V criteria (American Psychiatric Association, 2013). That is, a clinician specializing in stuttering evaluated each participant’s speech for dysfluencies such as repetition of sounds, prolongation of sounds, and blocking of sounds, and determined whether these dysfluencies interfered with communication. The presence of this last aspect is a requirement for the diagnosis of stuttering under the DSM-V. All participants were of Japanese ancestry; none of the participants had received any treatment for stuttering prior to or at the time of inclusion or any diagnosis of a psychiatric disorder, including social anxiety disorder. Four questionnaires (UTBAS-6-J, S-24-J, LSAS-J, and OASES-A-J) were administered before any interventions were conducted.

This study was conducted with the approval of the Ethics Committee of the Nippon Koukan Hospital (Approval number 201711). The study was designed and implemented according to the principles outlined in the Declaration of Helsinki (World Medical Association, 2013). We obtained the participants’ written informed consent before the study began. Every effort was made to protect the privacy and confidentiality of all participants.

### 2.2 Measures

#### 2.2.1 Japanese version of the brief unhelpful thoughts and beliefs about stuttering scale (UTBAS-6-J)

Since the original English version of the UTBAS has been translated into Japanese (UTBAS-J) and has been validated (Chu et al., 2017), and since the brief English version of the UTBAS (UTBAS-6) has been validated, we simply removed the same items from the UTBAS-J that were removed from the UTBAS to produce the UTBAS-6. Thus, this simple item-reduction process produced the Japanese version of the UTBAS-6 — the UTBAS-6-J. The UTBAS-6-J was subjected to psychometric analyses. We examined all items of UTBAS-6-J and reasoned that the six items of the UTBAS-6-J are culturally appropriate for Japanese speakers too. These six items are also found in the UTBAS-J, which was validated and assessed to be culturally relevant for Japanese speakers (Chu et al., 2017). As previously stated, higher scores on the UTBAS-6-J are indicative of a greater intensity and frequency of non-adaptive cognitions associated with stuttering.

#### 2.2.2 Modified Erickson Communication Attitude Scale – Japanese version (S-24-J)

The S-24-J is a questionnaire commonly used in clinical stuttering practice (Sasanuma and Ito, 1998; Iimura and Ishida, 2022). It consists of 24 questions to be answered with a “true” or “false” response. Items that are considered negative communication attitudes are summed and scored on a 24-point scale.

#### 2.2.3 Liebowitz Social Anxiety Scale – Japanese version (LSAS-J)

The LSAS-J is a screening test for social anxiety (Liebowitz, 1987; Asakura et al., 2002). Participants respond on a 4-point scale to each of 24 situations, indicating the extent of their fear and the degree to which they avoid the situations in question. The scores for “Fear “and “Avoidance” are summed to arrive at a sub-section score. The two sub-section scores are then summed to arrive at the total LSAS-J score.

#### 2.2.4 Overall Assessment of the Speaker’s Experience of Stuttering – Japanese version (OASES-A-J)

The OASES is a questionnaire designed to evaluate the experience of stuttering disorders from the perspective of the person who stutters (Yaruss and Quesal, 2006). It comprises 100 questions, which are answered on a 5-point scale, with negative responses resulting in higher scores. The questionnaire addresses four sections: (1) general perspectives about stuttering, (2) affective, behavioral, and cognitive reactions to stuttering, (3) functional communication difficulties, and (4) impact of stuttering on the speaker’s quality of life. For each section and in total, the mean score of the questionnaire items is calculated on a 5-point scale. The OASES-A is the OASES questionnaire for adults, and the OASES-A-J is the Japanese version of the OASES-A (Sakai et al., 2017).

### 2.3 Procedures

The UTBAS-6-J; S-24-J (Sasanuma and Ito, 1998); LSAS-J (Asakura et al., 2002); and OASES-A-J (Sakai et al., 2017) were administered to assess the reliability and validity of the UTBAS-6-J, as described below. To determine the internal consistency of the UTBAS-6-J, we calculated Cronbach’s alpha for the entire questionnaire; each of its sections (I. how frequently they have negative thoughts, II. how much they believe these thoughts, and III. how anxious these thoughts make them feel); and each of its items (1–6). Because the test–retest reliability of each item of the Japanese version of the UTBAS has already been confirmed (Chu et al., 2017) and because the items of the UTBAS-6-J are also contained within the UTBAS, we did not confirm the test–retest reliability of the UTBAS-6-J in this study.

To assess the concurrent validity of the UTBAS-6-J, we calculated Pearson’s correlation coefficient (r), using scores on the UTBAS-6-J and scores on the S-24-J, LSAS-J, and OASES-A-J. We also determined whether there was a correlation between UTBAS-6-J scores and participant age, and whether UTBAS-6-J scores differed by gender.

SPSS version 24 (IBM Corp, 2016) was used for statistical analyses; significance level was set to *p < 0.05*.

## 3 Results

### 3.1 Internal consistency of UTBAS-6-J

Table 1 presents summaries of the 56 PWS’ total and subsection scores on the UTBAS-6-J. Cronbach’s alpha for the UTABS-6-J total score was 0.947 (Table 1), which is considered excellent (Bland and Altman, 1997). Thus, the UTBAS-6-J has high internal consistency. Cronbach’s alphas for sections I, II, and III were 0.868, 0.881, and 0.926, respectively; all were slightly lower than that of the total score (0.947). However, considering the small number of items in each section, each section can also be considered to have sufficiently high internal consistency (Bland and Altman, 1997). All correlations between scores in each section were significantly positive (Pearson’s product–moment correlation, *r* = 0.584–0.787, *p* < 0.001) (Table 2).

**Table 1:** Summary of UTBAS-6-J baseline performance (*n* = 56).

	**Median**	**IQR**	**Cronbach’s alpha**	**Number of items**
UTBAS-6-J, Total score	47.0	35.0–61.8	0.947	18
UTBAS-6-J, Section I scores	16.0	11.8–22.0	0.868	6
UTBAS-6-J, Section II scores	14.0	9.0–18.5	0.881	6
UTBAS-6-J, Section III scores	17.5	12.0–24.0	0.926	6

The highest possible total score is 90, and the lowest possible total score is 18. Section I asks: “How FREQUENTLY I have these thoughts?” Section II asks: “How much I BELIEVE these thoughts?” Section III asks: “How ANXIOUS these thoughts make me feel?” IQR = interquartile range.

**Table 2:** Correlation (Pearson’s *r*) between sections of UTBAS-6-J.

	Section I	Section II	Section III
Section I	1.000	0.787*	0.755*
Section II	0.787*	1.000	0.584*
Section III	0.762*	0.534*	1.000

Pearson’s *r* in each two sections. *p < 0.001.

### 3.2 Concurrent validity of the UTBAS-6-J

**Figure 1:**
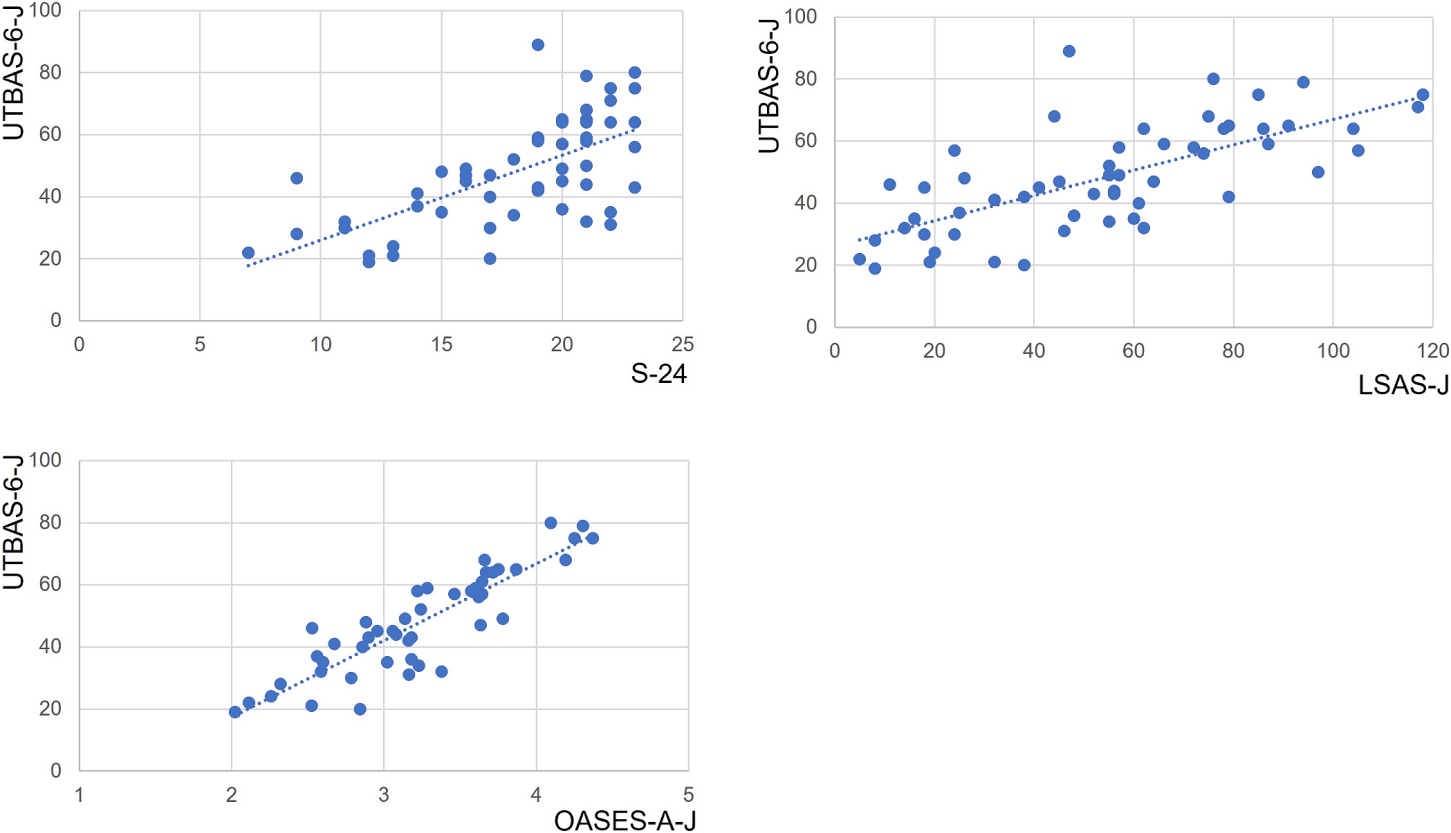
Correlations of UTBAS-6-J total scores with total scores of other questionnaires. UTBAS-6-J total scores significantly correlated with total scores on the OASES-A-J, the S-24, and the LSAS-J (all *p* < 0.001; Table 3). Dashed blue lines indicate the best fit linear regression line. LSAS-J, Liebowitz Social Anxiety Scale, Japanese version; OASES-A-J, Overall Assessment of the Speaker’s Experience of Stuttering, Japanese version; S-24-J, Modified Erickson Communication Attitude Scale, Japanese version.

We observed statistically significant correlations between the UTBAS-6-J and the S-24-J (*r* = 0.65, *p* < 0.01); LSAS-J (*r* = 0.70, *p* < 0.01); and OASES-A-J (*r* = 0.88, *p* < 0.01) (Table 3 and Figure 1). UTBAS-6-J scores were also significantly correlated with the subsection scores of the LSAS-J and the OASES-A-J (Table 3).

**Table 3:** Concurrent validity of the UTBAS-6-J, Pearson correlation.

	***r***	***p-*value**
S-24-J	0.65	<0.001
LSAS-J	0.70	<0.001
fear	0.66	<0.001
avoidance	0.69	<0.001
OASES-A-J	0.88	<0.001
Section 1	0.49	<0.001
Section 2	0.77	<0.001
Section 3	0.84	<0.001
Section 4	0.74	<0.001

LSAS-J, Liebowitz Social Anxiety Scale – Japanese version; OASES-A-J, Overall Assessment of the Speaker’s Experience of Stuttering – Japanese version; S-24-J, Modified Erickson Communication Attitude Scale – Japanese version.

### 3.3 Gender- and age-related differences in UTBAS-6-J scores

The UTBAS-6-J total scores of females were significantly higher than those of males (Mann–Whitney *U* test, *p* < 0.05; Table 4). Median scores on each section also tended to be higher in females. However, only the gender comparison of section II median scores reached statistical significance (Mann–Whitney *U* test, *p* < 0.05; Table 4).

**Table 4:** Comparison of UTBAS-6-J scores by gender.

**UTBAS-6-J**	**Male (*n* = 46)** **median (IQR)**	**Female (*n* = 10)** **median (IQR)**	**Mann–Whitney *U***	***P-*value**
Total	45.0 (34.3–58.8)	59.5 (47.5–76.3)	137	0.022
Section I	15.0 (11.0–21.8)	19.5 (14.5–23.5)	156	0.112
Section II	13.0 (9.0–17.0)	20.0 (11.5–24.0)	123.5	0.047
Section III	16.0 (11.3–21.8)	21.5 (17.3–28.5)	148.5	0.081

IQR, interquartile range.

There was no correlation between age and the UTBAS-6-J total score (Pearson’s product–moment correlation, *r* = −0.07, *p* = 0.63).

## 4 Discussion

Stuttering is universal (Van Riper, 1982; Yairi and Ambrose, 2013), occurring in all cultures and ethnic groups (Andrews et al., 1983; Zimmermann et al., 1983; Proctor et al., 2008; Zablotsky et al., 2019) to varying degrees. Stuttering in languages other than English has not been well studied (Ujihara et al., 2011). Regardless of the language, however, social anxiety is well-documented to be associated with stuttering (Craig and Tran, 2014; Iverach and Rapee, 2014; Khaled et al., 2022; Tomisato et al., 2022). The English version of the brief UTBAS-6 provides a reliable and efficient way to assess unhelpful thoughts and beliefs associated with speech-related anxiety in PWS (Iverach et al., 2016a). The aim of the present study was to evaluate whether the Japanese translation of the brief version of the UTBAS (UTBAS-6-J) is easy to administer to Japanese speakers in clinical and research settings. Another objective was to evaluate the concurrent validity of the UTBAS-6-J in light of other relevant assessment tools.

Our psychometric analysis of the UTBAS-6-J in 56 PWS revealed that its Cronbach’s alpha for internal consistency was 0.947. Thus, the UTBAS-6-J has high internal consistency, following the pattern of previous results for the original UTBAS, the Japanese version of the UTBAS, and the UTBAS-6 (St Clare et al., 2009; Iverach et al., 2016a; Chu et al., 2017) (Table 5). Furthermore, each section demonstrated a significant positive correlation with the others, indicating excellent internal consistency among the sections and the validity of summing section scores.

**Table 5:** Cronbach’s alpha values of different versions of the UTBAS.

**Version**	**Cronbach’s alpha**
UTBAS	0.96
UTBAS-J	0.94
UTBAS-6	0.82
UTBAS-6-J	0.947

Cronbach’s alphas for versions besides UTBAS-6-J come from St Clare et al. (2009), Iverach et al. (2016a,b), and Chu et al. (2017). UTBAS, Original Unhelpful Thoughts and Beliefs about Stuttering Scale (198 items); UTBAS-J, Japanese version of the UTBAS; UTBAS-6, Shortened version of the UTBAS (18 items); UTBAS-6-J, Japanese version of the UTBAS-6.

In the present study, the UTBAS-6-J also showed strong positive correlations with the S-24-J, the LSAS-J, and the OASES-A-J. The UTBAS-6-J measures non-adaptive cognition in stuttering. Those positive correlations mean that non-adaptive cognition was strongly related to negative communication attitude, social anxiety, and adverse impact of stuttering. The relationship between the original UTBAS and the State Trait Anxiety Inventory (STAI), the Social Avoidance and Distress Scale (SADS), and the Fear of Negative Evaluation Scale (FNE) has also been studied (St Clare et al., 2009). Findings from this previous research suggest that stuttering is associated with anxiety and fear. This is corroborated by findings of another study that the UTBAS-J is positively correlated with the S-24-J but negatively correlated with the SA scale (Chu et al., 2017). This suggests that the non-adaptive cognition of PWS is associated with their communication attitudes and speech performance (Chu et al., 2017). Taken together, non-adaptive cognition, as measured by the UTBAS-6 and UTBAS, are associated with psychological responses and behaviors associated with stuttering, such as anxiety. Thus, measuring non-adaptive cognition is important for deciding which interventions one might use for stuttering. The shortened version of the UTBAS-J (i.e., UTBAS-6-J) is capable of measuring non-adaptive cognition.

In the present study, female participants scored higher in the UTBAS-6-J than male participants, with total scale and section II scores reaching statistical significance. This trend, however, was not observed for the English version of the UTBAS-6 (Iverach et al., 2016a). One possible reason for this apparent disparity with Iverach et al.’s findings is that our study enrolled only a small number of women (*n* = 10). We also observed that female participants scored significantly higher in the OASES-A-J than male participants (data not shown), and we found that OASES-A-J and UTBAS-6-J scores were strongly and positively correlated. With the small sample size caveat, these results suggest that women were not any more likely to have non-adaptive cognition compared to men. Rather, the female participants in our study may have been more subjective than the male participants.

The current study has some limitations. First, as our participants were PWS who sought treatment for stuttering at a clinic, the results of this study cannot be simply generalized confidently to all PWS. The PWS who sought clinical treatment in our study represent a small proportion of PWS in Japan. Therefore, selection bias could have influenced our results because this subset of PWS might have been more motivated to seek treatment than other PWS in the community. Indeed, our subjects might have been more negatively affected by stuttering and social anxiety. Thus, our sample may have been biased. However, since the purpose of the UTBAS-6-J is to measure non-adaptive cognition associated with stuttering, the focus should be on identifying the kinds of non-adaptive cognition PWS who seek treatment may have, rather than identifying the kinds of non-adaptive cognition possessed by those who do not seek treatment. In the first place, the original UTBAS was based on the non-adaptive cognition of PWS who had received CBT (St Clare et al., 2009). Thus, when developing the UTBAS and creating its abbreviated version, the focus was on PWS who had sought treatment (Iverach et al., 2011, 2016a). Therefore, the authors reasoned that the UTBAS-6 should be consistent with PWS who have sought clinical treatment rather than that it be consistent with PWS in general. This consistency is demonstrated in the present study.

Second, the severity of objectively measured stuttering was not assessed, and thus was not included in the analysis. The severity of stuttering is typically measured objectively with a specific test, such as the %SS in the Stuttering Severity Instrument (Riley, 2009). Because objective severity was not measured consistently across participants in the present study, we did not include an analysis of severity of objective stuttering. However, we agree, it will be useful to examine the relationship between UTBAS-6-J scores and severity of objective stuttering. This aspect will be addressed and clarified in future studies. It is noteworthy that scores on questionnaires such as the S-24, LSAS, and OASES are well known *not* to correlate with objective severity of stuttering (Blumgart et al., 2012; Tomisato et al., 2022). In the present study, since UTBAS-6-J scores correlated strongly with S-24-J, LSAS-J, and OASES-A-J scores, UTBAS-6-J scores are expected *not* to correlate with severity of objective stuttering.

The UTBAS-6-J, the subject of the present study, is the Japanese version of the UTBAS-6. The UTBAS-6 was created with the intention of enabling the estimation of UTBAS scores from UTBAS-6 scores (Iverach et al., 2016a). The UTBAS-6-J was not designed as a questionnaire that allows for the inference of a UTBAS-J score from a UTBAS-6-J score. Additionally, the sample size was relatively small, comprising only 56 individuals. Future studies involving the administration of both the UTBAS-6-J and the full UTBAS-J to a large sample of PWS should be conducted to assess the convergence of scores on both short and long versions of the scale. Nevertheless, the results of the present study confirm the high validity of the UTBAS-6-J and demonstrate that it is a useful tool for measuring non-adaptive cognition in the clinical practice of stuttering.

## 5 Conclusion

The UTBAS-6-J is a useful short questionnaire for evaluating non-adaptive cognition in PWS whose native language is Japanese. Although the UTBAS and UTBAS-J provide a comprehensive assessment of non-adaptive thoughts related to stuttering and social anxiety, the shorter versions may be just as useful as the original longer versions, with the added benefit of being less time-consuming and burdensome to use in the clinic. The UTBAS-6-J has high internal consistency and correlates well with other relevant questionnaires.

## Data availability statement

The raw data supporting the conclusions of this article will be made available by the authors, without undue reservation.

## Ethics statement

The studies involving humans were approved by the Ethics Committee of the Nippon Koukan Hospital. The studies were conducted in accordance with the local legislation and institutional requirements. The participants provided their written informed consent to participate in this study.

## Author contributions

ST: Writing – original draft, Visualization, Resources, Project administration, Methodology, Investigation, Formal analysis, Data curation, Conceptualization. YY: Writing – review & editing, Validation, Resources, Investigation, Data curation. KW: Writing – review & editing, Methodology, Funding acquisition. TK: Writing – review & editing. HO: Writing – review & editing, Supervision.
