# Supplementary material for: Validity and utility of the Japanese version of the brief unhelpful thoughts and beliefs about stuttering scale: UTBAS-6-J
Source: Front Psychol. 2024 Jun 11;15:1382673. doi: 10.3389/fpsyg.2024.1382673 (PMC11196822; doi:10.3389/fpsyg.2024.1382673)
Supplement: Supplementary file 1 [file Table_1.DOC]

**短縮版吃音（きつおん・どもり）についての役に立たない考え・信念の評価尺度（UTBAS-6）**

|  | 下の6項目について、以下の3つの観点で、あなたにもっともよく当てはまる頻度や程度を選んで、そのスコア（**1**から**5**のどれか）を○で囲んでください。  **(1)** どのくらいしょっちゅう、そのように考える（思う）か。  **(2)** その考えをどのくらい信じているか（正しいと思うか）。  **(3)**（信じてなくても）もしそうだと考えたとすると（思ったとすると）、どの程度不安になるか。 | | | | | | | | | | | | | | | |
| --- | --- | --- | --- | --- | --- | --- | --- | --- | --- | --- | --- | --- | --- | --- | --- | --- |
|  | スコアのつけ方  **1**=決してない，全くない  **2**=稀に，少し  **3**=時々，やや  **4**=頻繁に，たくさん  **5**=いつも，完全に | (1)  どれくらいしょっちゅう、そのように考える（思う）か | | | | | (2)  その考えをどれくらい信じているか（正しいと思うか）。 | | | | | (3)  そうだと考えたとすると、どの程度不安になるか。 | | | | |
|  |  | 決して考えない | 稀にそう考える | 時々そう考える | しばしば考える | いつもそう考える | 全く違う | 少し正しい | やや正しい | だいたい正しい | 完全に正しい | 不安にならない | 少し不安になる | やや不安になる | 大いに不安 | 極めて不安 |
| 1 | 吃音（きつおん）があると、人生で本当に成功することは不可能だ。 | 1 | 2 | 3 | 4 | 5 | 1 | 2 | 3 | 4 | 5 | 1 | 2 | 3 | 4 | 5 |
| 2 | 私は吃る（どもる）ので、人々は私が無能であると思うだろう。 | 1 | 2 | 3 | 4 | 5 | 1 | 2 | 3 | 4 | 5 | 1 | 2 | 3 | 4 | 5 |
| 3 | 人々は、私が変人だと思うだろう。 | 1 | 2 | 3 | 4 | 5 | 1 | 2 | 3 | 4 | 5 | 1 | 2 | 3 | 4 | 5 |
| 4 | 私は行きたくない――私は人々に好いてもらえないだろう。 | 1 | 2 | 3 | 4 | 5 | 1 | 2 | 3 | 4 | 5 | 1 | 2 | 3 | 4 | 5 |
| 5 | 話そうとすること自体無駄だ――うまく言葉が出て来ないから。 | 1 | 2 | 3 | 4 | 5 | 1 | 2 | 3 | 4 | 5 | 1 | 2 | 3 | 4 | 5 |
| 6 | 私は用件を決して説明し終わらないだろう――誤解されるだろう。 | 1 | 2 | 3 | 4 | 5 | 1 | 2 | 3 | 4 | 5 | 1 | 2 | 3 | 4 | 5 |
